# Acute Kidney Injury Treated with Dialysis outside the Intensive Care Unit: A Retrospective Observational Single-Center Study

**DOI:** 10.1371/journal.pone.0163512

**Published:** 2016-09-27

**Authors:** Hannelore Sprenger-Mähr, Emanuel Zitt, Karl Lhotta

**Affiliations:** 1 Department of Nephrology and Dialysis, Academic Teaching Hospital Feldkirch, Feldkirch, Austria; 2 Vorarlberg Institute for Vascular Investigation and Treatment (VIVIT), Academic Teaching Hospital Feldkirch, Feldkirch, Austria; University of Messina, ITALY

## Abstract

**Introduction:**

The number of patients suffering from acute kidney injury requiring dialysis (AKI-D) is increasing. Whereas causes and outcome of AKI-D in the intensive care unit (ICU) are described extensively, few data exist about AKI-D patients treated outside the ICU. Aim of this study was to identify the causes of AKI-D, determine in-depth the comorbid conditions and outcome of this particular patient group and identify possibilities for its prevention.

**Methods:**

We retrospectively studied all AKI-D patients treated outside the ICU in a single nephrology referral center between January 2010 and June 2015. Data on comorbid conditions, renal function and drug therapy prior to AKI-D, and possible causal events were collected. Patients were grouped into those with renal hypoperfusion as the predominant cause of AKI-D (hemodynamic group) and those with other causes (non-hemodynamic group).

**Results:**

During 66 months 128 patients (57% male, mean age 69.3 years) were treated. AKI-D was community-acquired in 70.3%. The most frequent comorbidities were hypertension (62.5%), chronic kidney disease (CKD) (58.9%), coronary artery disease (CAD) (46.1%), diabetes (35.9%) and heart failure (34.1%). Most patients were prescribed diuretics (61.7%) and inhibitors of the renin-angiotensin-aldosterone system (RASI) (57.8%); 46.1% had a combination of both. In the 88 patients with hemodynamic AKI-D (68.8%) the most frequent initiating events were diarrhea (39.8%), infections (17.0%) and acute heart failure (13.6%). In the 40 patients with non-hemodynamic AKI-D (31.2%) interstitial nephritis (n = 15) was the prominent diagnosis. Patients with hemodynamic AKI-D were older (72.6 vs. 62.1 years, p = 0.001), suffered more often from CKD (68.2% vs. 33.3%, p = 0.003), CAD (54.5% vs. 27.5%, p = 0.004) and diabetes (42.0% vs. 22.5%, p = 0.033), and were more frequently on diuretics (75.0% vs. 32.5%, p<0.001), RASI (67.0% vs. 37.5%, p = 0.002) or their combination (58.0% vs. 20.0%, p<0.001). Twenty-two (17.2%) patients died and 27 (21.1%) patients died or developed end-stage renal disease.

**Conclusion:**

AKI-D treated outside the ICU is most often caused by renal hypoperfusion. It predominantly afflicts elderly patients with one or more comorbid conditions, who are treated with diuretics and RASI and have an acute illness leading to volume depletion. Early discontinuation of these drugs may be a successful strategy to avoid AKI-D in vulnerable patients.

## Introduction

The incidence of acute kidney injury (AKI) is increasing worldwide [1, 2]. AKI is associated with adverse outcomes such as chronic kidney disease (CKD), end-stage renal disease, hypertension or death and with a high economic burden [3–7]. In particular, patients suffering the most severe form of AKI requiring dialysis treatment (AKI-D) are at very high risk for death and, if surviving, for chronic or end-stage kidney disease [3, 8]. Several epidemiologic studies on AKI describe the incidence of all stages of AKI [9–11]. Other reports focus on patients with AKI-D, however without reporting whether patients were treated within or outside an intensive care unit (ICU) [12–16]. There is, however, a group of patients suffering from AKI-D, who do not need to be treated in an ICU. These patients are usually cared for at nephrology wards. Little is known about this particular group of patients. What are the causes of AKI-D, do they have predisposing comorbidities and cofactors such as medication, and what is their outcome? In an attempt to answer these questions, we performed a single-center retrospective observational study on all AKI-D patients treated at our nephrology department over a 66-month period. A better understanding of the clinical scenarios of pathomechanisms that typically lead to AKI-D treated outside the ICU may eventually enable clinicians to identify individuals at risk and prevent this serious condition.

In clinical practice and textbooks the terms prerenal, renal (including so-called acute tubular necrosis) and postrenal AKI are widely accepted. This classification is frequently misleading, because renal hypoperfusion will initially cause prerenal AKI, but when sustained and severe, cause acute tubular injury and renal AKI [17]. Many cases of AKI classified as renal AKI will actually be a consequence of renal hypoperfusion. Therefore, in our analysis we chose to divide cases in those in whom renal hypoperfusion was suspected to be the leading pathomechanism (hemodynamic AKI-D) and in those with other underlying causes (non-hemodynamic AKI-D).

## Subjects and Methods

### Study population

All adult patients with AKI-D treated in a single nephrology and dialysis unit at the Academic Teaching Hospital Feldkirch, a tertiary health care reference center serving a population of 400.000 inhabitants in the westernmost province of Austria, between January 2010 and June 2015 were evaluated retrospectively.

AKI-D was defined as AKI treated with acute intermittent hemodialysis. Dialysis was indicated at the discretion of the treating physicians. The team of nephrologists remained unchanged during the observation period. All patients with known end-stage renal disease and patients treated with renal replacement therapy in the ICU were excluded from this analysis.

In Austria retrospective studies do not require approval by an ethics committee. The local Ethics Committee of Vorarlberg confirmed that the study is exempt from ethics approval.

### Data sources

The clinical data obtained in that study were de-identified prior to analysis. All demographic characteristics, clinical data and laboratory values were retrieved from patient charts. Any AKI that had begun prior to hospitalization and was already diagnosed at admission was classified as community-acquired [18]. Any information on medications that might have contributed to the pathogenesis of AKI was collected, including the following classes of drugs: inhibitors of the renin-angiotensin-aldosterone system (RASI) such as ACE inhibitors (ACEI) angiotensin receptor blockers (ARB); loop diuretics and thiazide diuretics; non-steroidal anti-inflammatory drugs (NSAID); acetyl salicylic acid (ASS); metformin; proton pump inhibitors (PPI), antibiotics and statins. The following comorbidities were observed: hypertension, diabetes mellitus, congestive heart failure, coronary artery disease (CAD), peripheral artery disease (PAD), and liver failure. Smoking status (current smoker, non-smoker) was also retrieved from the patient chart. Preexisting serum creatinine values within the last 12 months before the actual hospitalization were documented when available from former hospital admissions and outpatient appointments (n = 90). A glomerular filtration rate estimated with the CKD-EPI formula (eGFR) <60 ml/min/1.73 m^2^ was defined as chronic kidney disease (CKD). Renal outcome (recovery of renal function or persistent dialysis dependence) was determined at discharge.

Special attention was paid to identify any acute illness that may have contributed to the pathogenesis of AKI. In our intention to detect the risk constellations for preventable AKI-D, we grouped all AKI-D into either hemodynamic or non-hemodynamic AKI-D. AKI-D was classified as hemodynamic when a decline in renal perfusion was considered the main cause and no other intrinsic renal disease could be found. All other cases with an intrinsic renal disease or another clear pathomechanism were classified as non-hemodynamic AKI-D. A consensus concerning classification was achieved amongst the three authors in all cases.

### Statistical analysis

Categorical variables are reported as absolute numbers (N) and percentages (%). Differences in frequencies between hemodynamic and non-hemodynamic AKI-D groups were compared using Pearson's χ^2^ test or Fisher's exact test, as appropriate. Continuous variables are presented as mean±standard deviation (SD). Univariate group comparisons for continuous variables were determined by unpaired Student's T test. A multivariable logistic regression analysis was calculated to estimate the risk for hemodynamic vs non-hemodynamic AKI-D associated the following variables: gender, age, RASI, loop diuretics. A two-sided p-value <0.05 was considered statistically significant. Statistical analyses were performed with IBM SPSS 22 (SPSS, Chicago, IL, USA).

## Results

During the observation period of 66 months a total of 128 patients (mean age 69.3±13.7 years, 43% women) with AKI-D were identified. The incidence per year remained stable with around 60 cases per million population. Of all cases 70.3% were community-acquired and 29.7% hospital-acquired. According to the causes of AKI-D 88 (68.7%) patients were classified as hemodynamic and 40 (31.3%) as non-hemodynamic. At diagnosis, 39.1% of the patients were anuric.

Table 1 shows demographic characteristics, comorbidities, medications and laboratory values for all patients, the hemodynamic and non-hemodynamic group. Patients with hemodynamic AKI-D were older (72.6 vs. 62.1 years, p = 0.001), more frequently women (48.9% vs. 30%, P = 0.046), suffered more often from CKD (68.2% vs. 33.3%, p = 0.003), CAD (54.5% vs. 27.5%, p = 0.004) or diabetes (42 vs. 22.5%, p = 0.033) and were more frequently treated with ACEI or ARB (67.0% vs. 37.5%, p = 0.002) and diuretics (75% vs. 32.5%, p<0.001) and had more impaired kidney function prior to AKI-D (creatinine 1.6 ± 0.8 vs. 1.1 ± 0.7 mg/dL, p = 0.018).

**Table 1:** Baseline characteristics (n = 128).

	N (%) or Mean±SD	Hemodynamic AKI-D, n = 88	Non-hemodynamic AKI-D, n = 40	P*
Gender, n(%)				0.046
Male	73 (57%)	45 (51.1%)	28 (70%)	
Female	55 (43%)	43 (48.9%)	12 (30%)	
Age (years)	69.3	72.6	62.1	0.001
Co-morbidities, n (%)				
Diabetes mellitus	46 (35.9%)	37 (42%)	9 (22.5%)	0.033
Hypertension	80 (62.5%)	58 (65.9%)	22 (55%)	0.237
Heart failure	24 (34.1%)	19 (21.6%)	5 (12.5%)	0.222
CAD	59 (46.1%)	48 (54.5%)	11 (27.5%)	0.004
PAD	30 (23.4%)	23 (26.1%)	7 (17.5%)	0.285
Liver cirrhosis	8 (6.3%)	6 (6.8%)	2 (5%)	0.999
Current smoker, n (%)	23 (18%)	13 (14.8%)	10 (25%)	0.162
Medication, n (%)				
ACE-I	47 (36.7%)	39 (44.3%)	8 (20%)	0.008
ARB	27 (21.1%)	20 (22.7%)	7 (17.5%)	0.502
ACE-I or ARB	74 (57.8%)	59 (67.0%)	15 (37.5%)	0.002
Loop diuretic	51 (39.8%)	44 (50.0%)	7 (17.5%)	< 0.001
Thiazide diuretic	47 (36.7%)	41 (46.6%)	6 (15.0%)	0.001
Loop or thiazide diuretic	79 (61.7%)	66 (75.0%)	13 (32.5%)	<0.001
Spironolactone	13 (10.2%)	11 (12.5%)	2 (5.0%)	0.343
NSAID	30 (23.4%)	19 (21.6%)	11 (27.5%	0.503
PPI	65 (50.8%)	44 (50.0%)	21 (52.5%)	0.793
Metformin	25 (19.5%)	21 (23.9%)	4 (10.0%)	0.092
Antibiotic	24 (18.8%)	17 (19.3%)	7 (17.5%)	0.807
Statin	60 (46.9%)	46 (52.3%)	14 (35.0%)	0.070
Aspirin	41 (32%)	33 (37.5%)	8 (20%)	0.049
Laboratory at diagnosis				
Creatinine (mg/dl)	6.8±2.8	6.6±2.8	7.1±2.9	0.321
Urea (mg/dl)	189.9±65.5	195.9±66.3	176.8±62.7	0.127
Potassium (mmol/L)	5.3±1.4	5.4±1.5	4.9±1.0	0.031
CRP (mg/dl)	8.5±10.5	8.5±10.9	8.4±9.8	0.964
Hb (g/L)	110.5±22.9	112.6±22.0	105.6±24.2	0.123
pH	7.32±0.12	7.3±0.1	7.4±0.1	<0.001
HCO_3_^-^ (mmol/L)	17.8±6.0	17.1±6.8	19.4±3.4	0.012
Creatinine (mg/dl) prior to AKI (n = 90)	1.5±0.8	1.6±0.8	1.1±0.7	0.018
eGFR (mL/min x 1.73m^3^) prior to AKI (n = 90)	54.6±26.1	49.2±24	69.7±26.2	<0.001
eGFR <60 ml/min x 1.73m^3^ prior to AKI (n = 90)	53 (58.9%)	45 (68.2%)	8 (33.3%)	0.003
Community acquired AKI	90 (70.3%)	63 (71.6%)	27 (67.5%)	0.639

Abbrevations: CAD, coronary artery disease; PAD, peripheral artery disease; +ACE-I, ACE-inhibitor; ARB, angiotensin II receptor blocker; NSAID, nonsteroidal anti-inflammatory drugs; PPI, proton pump inhibitors; CRP, C-reactive protein; Hb, hemoglobin; HCO_3_^-^, bicarbonate; AKI, acute kidney injury.

*group difference hemodynamic *vs* non-hemodynamic

Table 2 lists the main causes and initiating acute events of AKI-D for the hemodynamic and non-hemodynamic group. In hemodynamically-mediated cases diarrhea, infections and heart failure were the most frequent causes, whereas acute interstitial nephritis was the most frequent diagnosis in non-hemodynamic cases.

**Table 2:** Causes and initiating acute events of AKI-D.

Hemodynamic (n = 88)	N (%)	Non-hemodynamic (n = 40)	N (%)
Diarrhea	35 (39.8%)	Interstitial nephritis	15 (37.5%)
Septic diseases	15 (17.0%)	Rhabdomyolysis	5 (10%)
Pneumonia	5 (5.7%)		
Erysipelas	2 (2.3%)		
Critical limb ischemia	2 (2.3%)		
Cholangiosepsis	1 (1.1%)		
Gangrenous Cholecystitis	1 (1.1%)		
Urosepsis	3 (3.4%)		
Rectal abscess	1 (1.1%)		
Heart failure	12 (13.6%)	RPGN	4 (10%)
Dehydration	10 (8.8%)	Light-chain nephropathy/myeloma	3 (7.5%)
Hypotension	6 (6.8%)	Contrast-induced AKI	3 (7.5%)
Acute coronary syndrome	4 (4.6%)	Tumor lysis syndrome	2 (5.0%)
Blood loss	2 (2.3%)	Cholesterol emboli	2 (5.0%)
Hypercalcemia	2 (2.3%)	Ethylene glycol poisoning	2 (5.0%)
Decompensated liver cirrhosis	2 (2.3%)	Obstructive nephropathy	1 (2.5%)
		Catastrophic antiphospholipid syndrome	1 (2.5%)
		Cocaine/heroin intoxication	1 (2.5%)
		Warfarin-related nephropathy	1 (2.5%)

Abbrevations: RPGN, rapidly progressive glomerulonephritis; AKI, acute kidney injury.

Table 3 shows the combinations of drugs, comorbidities and causes in AKI-D patients.

**Table 3:** Exposure to variant medication combination causing AKI-D.

	All	Hemodynamic	Non-hemodynamic	P*
	N (%)	N (%)	N (%)	
Diuretics and RASI	59 (46.1)	51 (58%)	8 (20.0%)	<0.001
Diuretics and RASI and NSAID	12 (9.4%)	10 (11.4%)	2 (5.0%)	0.34
Diuretics and NSAID	16 (12.5%)	12 (13.6%)	4 (10.0%)	0.78
RASI and NSAID	17 (13.3%)	13 (14.8%)	4 (10.0%)	0.58

Abbrevations: NSAID, non- steroidal anti-inflammatory drugs.

*group difference hemodynamic *vs* non-hemodynamic

In the hemodynamic group 76 (86%) patients had at least a combination of two drugs, with the combination of a diuretic and a RASI being by far the most frequent (58%). The triple combination of diuretic, RASI and NSAID was found only in ten patients (11%). One-quarter of hemodynamic AKI-D was caused by the combination of diuretic, RASI and diarrhea, followed by the combination of diuretic, RASI and acute heart failure (8.7%) or infectious disease (6.8%).

In a multivariable logistic regression analysis the risk for hemodynamic vs non-hemodynamic AKI-D increased by 5% for each year of higher age (OR 1.05), by nearly 200% with pre-existing use of ACEI or ARB (OR 2.93) and by nearly 250% with preexisting loop diuretic therapy (OR 3.48) as shown in Table 4.

**Table 4:** Logistic regression analysis of risk factors for hemodynamic vs non-hemodynamic AKI-D.

Variable	Simple model		Multivariable model	
	OR (95% CI)	P	OR (95% CI)	P
Age (per year)	1.06 (1.03–1.10)	<0.001	1.05 (1.01–1.08)	0.015
Gender (1 = men, 0 = women)	0.45 (0.20–0.99)	0.048	0.44 (0.18–1.08)	0.073
RASI (1 = yes, 0 = no)	3.39 (1.56–7.39)	0.002	2.93 (1.23–6.98)	0.015
Loop diuretic (1 = yes, 0 = no)	4.71 (1.89–11.79)	0.001	3.48 (1.30–9.32)	0.013

Abbreviations: RASI, inhibitors of the renin-angiotensin-aldosterone system; OR, odds ratio; 95% CI, 95% confidence interval.

Overall, 101 patients (78.9%) survived with recovery of renal function (mean creatinine at discharge: 2.1±1.1 mg/dL). The median number of dialysis sessions was three (25^th^-75^th^ percentile: 2–6) with a maximum of 18. Patients classified as suffering from hemodynamic AKI-D needed a smaller number of dialysis sessions until recovery of renal function (3 vs. 5 sessions, p = 0.002). Twenty-two (17.2%) patients died and 27 (21.1%) patients died or developed end-stage renal disease requiring chronic dialysis. Patients without renal recovery were older (75.1 vs. 67.9 years, p = 0.018), had slightly more impaired kidney function prior to AKI-D (creatinine 1.7±1.0 vs. 1.4±0.7 mg/dL, p = 0.061), more often suffered from CAD (64% vs. 41.7%, p = 0.045) and were more frequently treated with loop diuretics prior to AKI-D (64% vs. 34%, p = 0.006). These outcome parameters did not differ between hemodynamic and non-hemodynamic AKI-D (Table 5).

**Table 5:** Renal and patient outcome.

	Non-hemodynamic	Hemodynamic	P
Recovery of kidney function	34 (85.0%)	69 (78.4%)	0.383
Death	5 (12.5%)	17 (19.3%)	0.343
Dialysis or death	7 (17.5%)	20 (22.7%)	0.502

## Discussion

To the best of our knowledge, there are no or only limited data on the group of AKI-D patients treated outside an ICU. Therefore, this retrospective single-center study of 128 patients with this condition provides several important new findings. Nearly 70% of AKI-D was community-acquired. Data on whether AKI-D, treated either inside or outside the ICU, is community- or hospital-acquired, are lacking. Our results are comparable to the 67% of community-acquired AKI observed in district general hospitals [10], but higher than the 30% found in a large university teaching hospital [11]. However, both studies included all stages of AKI and the differences are probably due to hospital type.

One-third of our patients with AKI-D were classified as non-hemodynamic. These patients were on average ten years younger, had fewer comorbidities and were less frequently treated with RASI and diuretics. Interstitial nephritis, rapidly progressive glomerulonephritis and crush kidney were the predominant diagnoses. NSAID exposure did not differ between the two groups. Whereas NSAIDs were implicated in the disruption of renal perfusion in the hemodynamic group, they were identified as underlying drugs in cases of acute interstitial nephritis in the non-hemodynamic group. NSAIDs have been described as a frequent cause of acute interstitial nephritis [19, 20]. Overall, it seems that non-hemodynamic AKI-D is predominantly caused by conditions that affect renal tubular function.

The other two-thirds of the cases were classified as predominantly hemodynamically mediated. This contrasts with AKI-D patients admitted to the ICU, in whom sepsis with multi-organ failure is the most frequent cause of AKI [13, 21]. In addition, patients with AKI-D treated outside the ICU most frequently acquired AKI outside the hospital. Although data are lacking, we believe that most patients treated in the ICU have hospital-acquired AKI-D. AKI-D is on the rise [14–16]. A recent study on AKI-D found, in addition to showing an increase of 11% per year between 2007 and 2009, that this increase was due to septicemia, hypertension, respiratory failure, hemorrhagic shock and liver disease, and not due to surgeries or other therapeutic interventions [22]. It was not stated whether these patients had community- or hospital-acquired AKI-D and were treated in or outside the ICU. Our data suggest that in patients treated outside the ICU, a combination of underlying comorbid conditions, concurrent medication affecting renal perfusion and an acute illness will precede hemodynamically mediated AKI-D in the vast majority of patients.

Among underlying conditions CKD, hypertension and CAD were most frequently observed, followed by diabetes, heart failure and PAD. The relative frequency of comorbidities was comparable to [23] or much higher [13, 15, 16] than in other AKI-D cohorts. Many of our patients had two or more comorbidities. In addition, the patients with hemodynamic AKI-D were on average ten years older compared to patients with non-hemodynamic AKI-D and other AKI-D cohorts [14, 16]. That means we are dealing with a particularly vulnerable population.

Whereas in our total population and in other reports a 60% male predominance in AKI-D cases was found ([13, 15, 16, 23], this gender difference interestingly disappeared when focusing on hemodynamic AKI-D.

A second important component in the pathogenesis of hemodynamic AKI-D seems to be the concurrent use of drugs that can impair renal perfusion in the presence of concomitant volume contraction. Out of these drugs, RASI and diuretics are standard of care in treatment of the comorbidities found in our patients such as CKD, hypertension, CAD, diabetes or heart failure. The most frequently used medications in our patients were diuretics with loop diuretics and thiazides in equal frequency. Diuretics have been described as the most frequent drugs involved in the pathogenesis of AKI, prescribed in 50% of nursing home residents developing AKI [24]. In our retrospective study 74 (57.8%) patients were treated with ACEI or ARB, especially in the group with hemodynamically induced AKI-D. A recent study from England showed an annual increase of 15.8% in RASI prescriptions between 2007 and 2011 with a concomitant 52% rise in AKI hospital admissions [25]. The authors conclude that 15% of the increase in AKI admissions could have been avoided if the RASI prescription rate had remained constant.

The use of NSAIDs is not only associated with chronic kidney disease [26], but also another risk factor for drug-induced AKI, because NSAIDs cause afferent arteriolar vasoconstriction through inhibition of prostaglandin synthesis. The use of NSAIDs alone is relatively harmless [27]. Combined therapy with RASI and especially with diuretics is associated with a significant increase in AKI [28–30]. The triple combination of NSAIDs with RASI and diuretics, a so-called “triple whammy,” seems to be the most deleterious one [29, 31, 32]. Patients with chronic renal impairment, using loop diuretics and over the age of 75 years are at greatest risk [29]. These data are in line with our findings. In our cohort of hemodynamically mediated AKI-D 86% of the patients had a combination therapy of at least two of the above-mentioned drugs, with the diuretic and RASI combination being most frequent, whereas only a minority of our AKI-D cases was due to the “triple whammy”.

The third and possibly most critical step in the development of AKI-D in a vulnerable patient is an acute intercurrent illness, which was present in all 88 patients with hemodynamic AKI-D. The predominant acute injuries were diarrhea with volume depletion, infectious diseases, acute heart failure and dehydration caused by other factors. What is particularly striking in our cohort is the very high frequency (40%) of diarrhea as a precipitating event for hemodynamic AKI-D. Of note, surgery or medical procedures, reported in other studies as main causes of AKI-D [12, 22], were (except for three cases of contrast-induced AKI) not observed in our cohort.

How can this common sequence of underlying comorbidities, drug therapy and acute precipitating event leading to AKI-D be interrupted?

One option is the early and judicious correction of volume depletion in order to improve systemic and renal perfusion [17]. Fluid overload, however, has to be avoided in AKI patients, because it may cause interstitial edema with organ dysfunctions and increase mortality [33, 34].

A second attractive option would be to discontinue diuretics, RASI and NSAR in elderly patients with co-morbidities such as CKD, hypertension, CAD and diabetes in the setting of an acute illness that may cause volume depletion.

KDIGO recommends interrupting RASI in CKD patients in the case of inter-current illnesses that lead to dehydration as a result of diarrhea, vomiting or high fever [35]. According to British National Institute for Health and Care Excellence (NICE) guidelines RASI should be temporarily stopped in all patients with diarrhea, vomiting or sepsis until their clinical condition has improved and stabilized [36]. Our results suggest that not only RASI but also diuretics should be withheld in high-risk situations. In most instances these simple measures have to be taken outside the hospital setting. This requires information and education of patients, their care-givers and general practitioners. Whether the general implementation of such “sick day rules” can reduce the incidence of AKI-D or can even cause harm is presently unknown. Therefore, such programs should be accompanied by a scientific evaluation in order to prove their efficacy or identify possible negative consequences of pausing potent drugs [37]. Although our results suggest that pausing hemodynamically active drugs may be beneficial in certain instances, due to the observational design of our study no conclusions can be made as to whether stopping any medications would have prevented the AKI in our patients.

Even a minor acute deterioration of kidney function dramatically increases the risk for death in hospitalized patients [7, 38]. In AKI-D patients treated in the ICU mortality rates between 40% and 60% are reported [12, 13, 39, 40]. Mortality of patients not admitted to the ICU is probably lower. A study from Italy reported a mortality rate of of 41.2% in AKI-D patients treated outside the ICU and of 65.5% in those treated at the ICU [23].

Twenty-two (17.2%) patients from our study population died and 27 (21.1%) patients died or remained on dialysis. This more favorable outcome could be explained by the underlying cause of AKI-D due to a combination effect of drugs and volume depletion. This may allow more rapid recovery of kidney function as shown by the small number of dialysis treatments and concomitant lower mortality and ESRD rate.

Our study is limited by its small number of patients and retrospective design.

The strategies to admit a patient to the ICU or to use intermittent or continuous renal replacement therapy may differ between institutions and therefore our selection of AKI-D patients treated outside the ICU with hemodialysis may not be generally applicable.

As AKI is usually multifactorial, classification as hemodynamic or non-hemodynamic is not always clear-cut. Although a good agreement was reached amongst the authors, this classification remains nevertheless subjective. This may be particularly relevant to contrast-induced AKI, which results from a combination of hemodynamic and toxic insults to the kidney. The three cases of contrast-induced AKI-D in our study population were all hospital-based and developed after percutaneous coronary intervention in patients with acute coronary syndrome, who were treated with a RASI and a diuretic. Hypotension and heart failure are the most important risk factors for AKI after coronary angiography and intravenous volume expansion is the most effective intervention for its prevention [41]. In addition, a specific risk score, which includes age, serum creatinine and ejection fraction (ACEF score), is able to predict contrast-induced AKI after percutaneous coronary intervention [42]. After careful evaluation of the patient records we concluded that these cases were not caused primarily by volume depletion and renal hypoperfusion but by the toxic effect of contrast media and we therefore classified them as non-hemodynamic. We acknowledge, however, that this classification remains arbitrary.

Furthermore, data on preexisting proteinuria, which is a potent risk factor for AKI, are lacking.

The particular strength of the study is that our data relied on the original and complete patient charts, which allowed in-depth determination of comorbidities, concurrent drug therapies and inter-current acute illness. In this single-center study all patients were treated by the same physicians and the indication for commencement of hemodialysis therapy was established by one and the same medical team over the entire term following current best practice guidelines.

## Conclusion

AKI-D outside the ICU is caused mainly by a combination of underlying comorbidities, drug therapy and volume depletion with decreased renal perfusion. Discontinuing diuretics, RASI and NSAID in the case of an acute illness may be a fruitful approach to avoiding AKI-D in vulnerable patients.
